# Trichlorido(4-methyl­benz­yl)bis­(1*H*-pyrazole-κ*N* ^2^)tin(IV)

**DOI:** 10.1107/S1600536811015698

**Published:** 2011-05-07

**Authors:** Thy Chun Keng, Kong Mun Lo, Seik Weng Ng

**Affiliations:** a Department of Chemistry, University of Malaya, 50603 Kuala Lumpur, Malaysia

## Abstract

The six-coordinate Sn^IV^ atom in the title compound, [Sn(C_8_H_9_)Cl_3_(C_3_H_4_N_2_)_2_], shows an octa­hedral coordination. The N atoms of the N-heterocycle are *cis* to each other. The Sn—N bond that is *trans* to the Sn—C bond is shorter than the Sn—N bond *trans* to the Sn—Cl bond. Weak N—H⋯Cl hydrogen bonds link adjacent mol­ecules, generating a double chain running along the *c* axis.

## Related literature

For the direct synthesis of the organotin chloride reactant, see: Sisido *et al.* (1961 ▶). For the trichloridophenyl­tin–di(pyrazole) adduct, see: Casas *et al.* (1996 ▶).
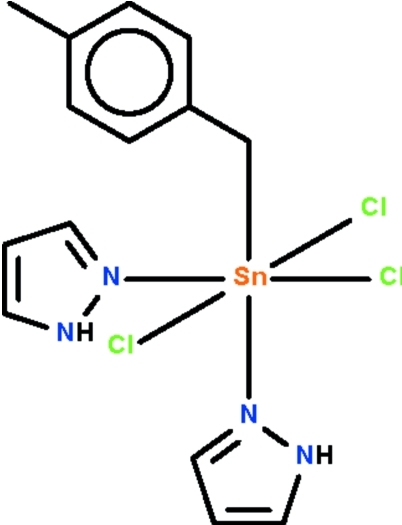


## Experimental

### 

#### Crystal data

[Sn(C_8_H_9_)Cl_3_(C_3_H_4_N_2_)_2_]
*M* *_r_* = 466.36
Monoclinic, 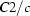

*a* = 34.7322 (4) Å
*b* = 7.3709 (1) Å
*c* = 14.5760 (2) Å
β = 109.0535 (5)°
*V* = 3527.13 (8) Å^3^
*Z* = 8
Mo *K*α radiation
μ = 1.90 mm^−1^
*T* = 100 K
0.30 × 0.25 × 0.20 mm

#### Data collection

Bruker SMART APEX diffractometer
Absorption correction: multi-scan (*SADABS*; Sheldrick, 1996 ▶) *T*_min_ = 0.599, *T*_max_ = 0.702
16131 measured reflections
4053 independent reflections
3734 reflections with *I* > 2σ(*I*)
*R*_int_ = 0.022

#### Refinement

*R*[*F* ^2^ > 2σ(*F* ^2^)] = 0.017
*wR*(*F* ^2^) = 0.044
*S* = 1.00
4053 reflections
208 parameters
2 restraints
H atoms treated by a mixture of independent and constrained refinement
Δρ_max_ = 0.43 e Å^−3^
Δρ_min_ = −0.26 e Å^−3^

### 

Data collection: *APEX2* (Bruker, 2009 ▶); cell refinement: *SAINT* (Bruker, 2009 ▶); data reduction: *SAINT*; program(s) used to solve structure: *SHELXS97* (Sheldrick, 2008 ▶); program(s) used to refine structure: *SHELXL97* (Sheldrick, 2008 ▶); molecular graphics: *X-SEED* (Barbour, 2001 ▶); software used to prepare material for publication: *publCIF* (Westrip, 2010 ▶).

## Supplementary Material

Crystal structure: contains datablocks global, I. DOI: 10.1107/S1600536811015698/bt5524sup1.cif

Structure factors: contains datablocks I. DOI: 10.1107/S1600536811015698/bt5524Isup2.hkl

Additional supplementary materials:  crystallographic information; 3D view; checkCIF report

## Figures and Tables

**Table 1:** Hydrogen-bond geometry (Å, °)

*D*—H⋯*A*	*D*—H	H⋯*A*	*D*⋯*A*	*D*—H⋯*A*
N2—H2⋯Cl1^i^	0.87 (1)	2.56 (2)	3.265 (2)	139 (2)
N4—H4⋯Cl1^ii^	0.88 (1)	2.65 (2)	3.270 (2)	129 (2)

Symmetry codes: (i) 
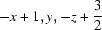
; (ii) 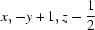.

**Fig. 1.**
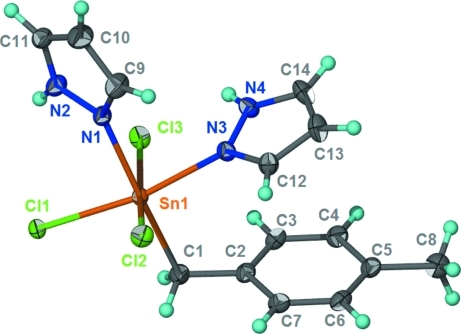
Anisotropic displacement ellipsoid plot (Barbour, 2001) of SnCl3(C8H9)(C3H4N2)2 at the 70% probability level; hydrogen atoms are drawn as spheres of arbitrary radius.

## supplementary crystallographic information

### Comment

Dibenzyltin dichloride and benzyltin trichloride can be synthesized by the direct action of benzyl chloride on stannous chloride; other ring-substituted analogs are similarly synthesized (Sisido *et al.*, 1961). The title compound results from the reaction of di(4-methylbenzyl)tin dichloride with pyrazole to afford the pyrazole adduct of a monoorganotin trichloride. There are few examples of monororganotin chlorides forming adducts with *N*-heterocycles. Phenyltin trichloride forms a 1:2 adduct with pyrazole (Casas *et al.*, 1996). The 4-methylbenzyl analog affords a similar 1:2 adduct. The six-coordinate Sn^IV^ atom in SnCl_3_(C_8_H_9_)(C_3_H_4_N_2_)_2_ (Scheme I) shows octahedral coordination. The N atoms of the *N*-heterocycle are *cis* to each other and the three Cl atoms are coplanar (Fig. 1). The geometry can be described as being a *mer*-octahedron. The Sn–N bond that is *trans* to the Sn–C bond is shorter than the Sn–N bond *trans* to the Sn–Cl bond.

### Experimental

Di(4-methylbenzyl)tin dichloride was synthesized by using a literature procedure (Sisido *et al.*, 1961). The compound (0.4 g, 1 mmol) and pyrazole (0.136 g, 2 mmol) of pyrazole were dissolved in ethanol (100 ml) and the solution was heated for an hour. The solution was filtered and then set aside for the growth of colorless crystals.

### Refinement

Carbon-bound H-atoms were placed in calculated positions (C—H 0.95 to 0.99 Å) and were included in the refinement in the riding model approximation, with *U*(H) set to 1.2 times *U*_eq_(C). The amino H-atoms were located in a difference Fourier map, and were refined isotropically with a distance restraint of N–H 0.88±0.01 Å.

### Crystal data

**Table:**

[Sn(C_8_H_9_)Cl_3_(C_3_H_4_N_2_)_2_]	*F*(000) = 1840
*M**_r_* = 466.36	*D*_x_ = 1.756 Mg m^−^^3^
Monoclinic, *C*2/*c*	Mo *K*α radiation, λ = 0.71073 Å
Hall symbol: -C 2yc	Cell parameters from 9748 reflections
*a* = 34.7322 (4) Å	θ = 2.5–28.3°
*b* = 7.3709 (1) Å	µ = 1.90 mm^−^^1^
*c* = 14.5760 (2) Å	*T* = 100 K
β = 109.0535 (5)°	Block, colorless
*V* = 3527.13 (8) Å^3^	0.30 × 0.25 × 0.20 mm
*Z* = 8	

### Data collection

**Table:**

Bruker SMART APEX diffractometer	4053 independent reflections
Radiation source: fine-focus sealed tube	3734 reflections with *I* > 2σ(*I*)
graphite	*R*_int_ = 0.022
ω scans	θ_max_ = 27.5°, θ_min_ = 2.5°
Absorption correction: multi-scan (*SADABS*; Sheldrick, 1996)	*h* = −44→44
*T*_min_ = 0.599, *T*_max_ = 0.702	*k* = −9→9
16131 measured reflections	*l* = −18→18

### Refinement

**Table:**

Refinement on *F*^2^	Primary atom site location: structure-invariant direct methods
Least-squares matrix: full	Secondary atom site location: difference Fourier map
*R*[*F*^2^ > 2σ(*F*^2^)] = 0.017	Hydrogen site location: inferred from neighbouring sites
*wR*(*F*^2^) = 0.044	H atoms treated by a mixture of independent and constrained refinement
*S* = 1.00	*w* = 1/[σ^2^(*F*_o_^2^) + (0.022*P*)^2^ + 3.9233*P*] where *P* = (*F*_o_^2^ + 2*F*_c_^2^)/3
4053 reflections	(Δ/σ)_max_ = 0.001
208 parameters	Δρ_max_ = 0.43 e Å^−^^3^
2 restraints	Δρ_min_ = −0.26 e Å^−^^3^

### Fractional atomic coordinates and isotropic or equivalent isotropic displacement parameters (Å^2^)

**Table:**

	*x*	*y*	*z*	*U*_iso_*/*U*_eq_	
Sn1	0.595182 (3)	0.582348 (14)	0.721693 (7)	0.01211 (4)	
Cl1	0.555981 (12)	0.42823 (5)	0.81341 (3)	0.01619 (8)	
Cl2	0.626900 (12)	0.79953 (6)	0.85059 (3)	0.01902 (9)	
Cl3	0.556206 (12)	0.41810 (5)	0.57311 (3)	0.01787 (9)	
N1	0.54480 (4)	0.77932 (19)	0.67420 (10)	0.0150 (3)	
N2	0.50505 (4)	0.7311 (2)	0.64558 (11)	0.0183 (3)	
H2	0.4985 (7)	0.6177 (16)	0.6474 (18)	0.038 (7)*	
N3	0.62026 (4)	0.74995 (19)	0.62547 (10)	0.0153 (3)	
N4	0.60725 (4)	0.7316 (2)	0.52785 (10)	0.0166 (3)	
H4	0.5873 (5)	0.657 (3)	0.4997 (15)	0.031 (6)*	
C1	0.64493 (5)	0.3890 (2)	0.76417 (13)	0.0179 (3)	
H1A	0.6574	0.3912	0.8357	0.021*	
H1B	0.6339	0.2657	0.7453	0.021*	
C2	0.67715 (5)	0.4263 (2)	0.71934 (13)	0.0161 (3)	
C3	0.67158 (5)	0.3774 (2)	0.62338 (13)	0.0172 (3)	
H3	0.6472	0.3172	0.5866	0.021*	
C4	0.70114 (5)	0.4155 (2)	0.58085 (13)	0.0192 (4)	
H4A	0.6967	0.3807	0.5154	0.023*	
C5	0.73723 (5)	0.5037 (2)	0.63237 (13)	0.0179 (3)	
C6	0.74257 (5)	0.5557 (2)	0.72790 (13)	0.0181 (3)	
H6	0.7667	0.6179	0.7642	0.022*	
C7	0.71300 (5)	0.5175 (2)	0.77065 (12)	0.0174 (3)	
H7	0.7173	0.5539	0.8357	0.021*	
C8	0.76965 (6)	0.5415 (3)	0.58673 (14)	0.0252 (4)	
H8A	0.7843	0.6527	0.6145	0.038*	
H8B	0.7569	0.5566	0.5165	0.038*	
H8C	0.7888	0.4397	0.5995	0.038*	
C9	0.54488 (6)	0.9595 (2)	0.66532 (15)	0.0234 (4)	
H9	0.5687	1.0325	0.6800	0.028*	
C10	0.50533 (6)	1.0249 (3)	0.63159 (15)	0.0255 (4)	
H10	0.4970	1.1478	0.6191	0.031*	
C11	0.48082 (5)	0.8757 (2)	0.62004 (12)	0.0187 (3)	
H11	0.4519	0.8751	0.5979	0.022*	
C12	0.65211 (5)	0.8626 (2)	0.64665 (13)	0.0177 (3)	
H12	0.6676	0.9003	0.7103	0.021*	
C13	0.65924 (6)	0.9165 (2)	0.56243 (14)	0.0226 (4)	
H13	0.6799	0.9963	0.5572	0.027*	
C14	0.63001 (6)	0.8300 (2)	0.48815 (13)	0.0215 (4)	
H14	0.6266	0.8385	0.4209	0.026*	

### Atomic displacement parameters (Å^2^)

**Table:**

	*U*^11^	*U*^22^	*U*^33^	*U*^12^	*U*^13^	*U*^23^
Sn1	0.01177 (6)	0.01333 (6)	0.01165 (6)	−0.00092 (4)	0.00438 (4)	−0.00068 (4)
Cl1	0.01552 (18)	0.01766 (19)	0.01712 (19)	0.00114 (14)	0.00767 (15)	0.00354 (15)
Cl2	0.01874 (19)	0.0214 (2)	0.01536 (18)	−0.00281 (15)	0.00349 (15)	−0.00550 (15)
Cl3	0.0199 (2)	0.01818 (19)	0.01524 (19)	−0.00530 (15)	0.00538 (15)	−0.00409 (15)
N1	0.0132 (6)	0.0161 (7)	0.0157 (7)	−0.0021 (5)	0.0047 (5)	−0.0007 (5)
N2	0.0138 (7)	0.0173 (7)	0.0225 (7)	−0.0023 (6)	0.0041 (6)	−0.0002 (6)
N3	0.0163 (6)	0.0172 (7)	0.0124 (6)	−0.0017 (5)	0.0048 (5)	−0.0013 (5)
N4	0.0196 (7)	0.0167 (7)	0.0134 (6)	−0.0020 (6)	0.0052 (6)	−0.0002 (6)
C1	0.0167 (8)	0.0177 (8)	0.0199 (8)	0.0016 (6)	0.0070 (7)	0.0023 (7)
C2	0.0152 (8)	0.0142 (8)	0.0194 (8)	0.0023 (6)	0.0063 (6)	0.0007 (6)
C3	0.0136 (7)	0.0172 (8)	0.0204 (8)	0.0005 (6)	0.0047 (6)	−0.0030 (7)
C4	0.0191 (8)	0.0215 (9)	0.0178 (8)	0.0032 (7)	0.0071 (7)	−0.0022 (7)
C5	0.0156 (8)	0.0167 (8)	0.0225 (9)	0.0033 (6)	0.0077 (7)	0.0029 (7)
C6	0.0137 (8)	0.0160 (8)	0.0231 (9)	0.0006 (6)	0.0039 (7)	0.0002 (7)
C7	0.0174 (8)	0.0181 (8)	0.0157 (8)	0.0022 (6)	0.0042 (6)	−0.0012 (7)
C8	0.0208 (9)	0.0311 (10)	0.0270 (10)	0.0004 (8)	0.0124 (8)	0.0025 (8)
C9	0.0198 (9)	0.0155 (8)	0.0351 (10)	−0.0020 (7)	0.0090 (8)	0.0019 (8)
C10	0.0234 (9)	0.0171 (8)	0.0358 (11)	0.0039 (7)	0.0095 (8)	0.0055 (8)
C11	0.0160 (8)	0.0226 (9)	0.0174 (8)	0.0026 (7)	0.0055 (7)	−0.0001 (7)
C12	0.0173 (8)	0.0168 (8)	0.0193 (8)	−0.0028 (6)	0.0066 (7)	−0.0010 (7)
C13	0.0270 (9)	0.0193 (9)	0.0266 (10)	−0.0049 (7)	0.0156 (8)	−0.0012 (7)
C14	0.0319 (10)	0.0176 (9)	0.0193 (8)	−0.0008 (7)	0.0141 (8)	0.0019 (7)

### Geometric parameters (Å, °)

**Table:**

Sn1—C1	2.1680 (17)	C4—C5	1.394 (2)
Sn1—N1	2.2042 (14)	C4—H4A	0.9500
Sn1—N3	2.2471 (14)	C5—C6	1.397 (2)
Sn1—Cl2	2.4402 (4)	C5—C8	1.509 (2)
Sn1—Cl3	2.4646 (4)	C6—C7	1.393 (2)
Sn1—Cl1	2.4739 (4)	C6—H6	0.9500
N1—C9	1.334 (2)	C7—H7	0.9500
N1—N2	1.3528 (19)	C8—H8A	0.9800
N2—C11	1.333 (2)	C8—H8B	0.9800
N2—H2	0.869 (10)	C8—H8C	0.9800
N3—C12	1.336 (2)	C9—C10	1.386 (3)
N3—N4	1.3518 (19)	C9—H9	0.9500
N4—C14	1.336 (2)	C10—C11	1.368 (3)
N4—H4	0.876 (9)	C10—H10	0.9500
C1—C2	1.494 (2)	C11—H11	0.9500
C1—H1A	0.9900	C12—C13	1.388 (2)
C1—H1B	0.9900	C12—H12	0.9500
C2—C3	1.395 (2)	C13—C14	1.376 (3)
C2—C7	1.398 (2)	C13—H13	0.9500
C3—C4	1.390 (2)	C14—H14	0.9500
C3—H3	0.9500		
			
C1—Sn1—N1	178.30 (6)	C2—C3—H3	119.5
C1—Sn1—N3	96.00 (6)	C3—C4—C5	121.27 (16)
N1—Sn1—N3	82.62 (5)	C3—C4—H4A	119.4
C1—Sn1—Cl2	95.39 (5)	C5—C4—H4A	119.4
N1—Sn1—Cl2	85.54 (4)	C6—C5—C4	117.92 (16)
N3—Sn1—Cl2	87.15 (4)	C6—C5—C8	120.91 (16)
C1—Sn1—Cl3	94.93 (5)	C4—C5—C8	121.17 (16)
N1—Sn1—Cl3	84.01 (4)	C5—C6—C7	120.88 (16)
N3—Sn1—Cl3	86.29 (4)	C5—C6—H6	119.6
Cl2—Sn1—Cl3	168.293 (15)	C7—C6—H6	119.6
C1—Sn1—Cl1	94.11 (5)	C2—C7—C6	121.07 (16)
N1—Sn1—Cl1	87.23 (4)	C2—C7—H7	119.5
N3—Sn1—Cl1	169.61 (4)	C6—C7—H7	119.5
Cl2—Sn1—Cl1	94.297 (14)	C5—C8—H8A	109.5
Cl3—Sn1—Cl1	90.458 (14)	C5—C8—H8B	109.5
C9—N1—N2	105.40 (14)	H8A—C8—H8B	109.5
C9—N1—Sn1	131.25 (12)	C5—C8—H8C	109.5
N2—N1—Sn1	123.34 (11)	H8A—C8—H8C	109.5
C11—N2—N1	111.34 (14)	H8B—C8—H8C	109.5
C11—N2—H2	128.9 (16)	N1—C9—C10	110.35 (16)
N1—N2—H2	119.7 (16)	N1—C9—H9	124.8
C12—N3—N4	105.80 (13)	C10—C9—H9	124.8
C12—N3—Sn1	131.13 (11)	C11—C10—C9	105.59 (16)
N4—N3—Sn1	122.52 (10)	C11—C10—H10	127.2
C14—N4—N3	111.11 (14)	C9—C10—H10	127.2
C14—N4—H4	129.0 (15)	N2—C11—C10	107.31 (15)
N3—N4—H4	119.8 (15)	N2—C11—H11	126.3
C2—C1—Sn1	113.26 (11)	C10—C11—H11	126.3
C2—C1—H1A	108.9	N3—C12—C13	110.31 (15)
Sn1—C1—H1A	108.9	N3—C12—H12	124.8
C2—C1—H1B	108.9	C13—C12—H12	124.8
Sn1—C1—H1B	108.9	C14—C13—C12	105.33 (16)
H1A—C1—H1B	107.7	C14—C13—H13	127.3
C3—C2—C7	117.93 (15)	C12—C13—H13	127.3
C3—C2—C1	120.81 (15)	N4—C14—C13	107.45 (16)
C7—C2—C1	121.21 (15)	N4—C14—H14	126.3
C4—C3—C2	120.92 (16)	C13—C14—H14	126.3
C4—C3—H3	119.5		
			
N3—Sn1—N1—C9	−43.97 (16)	Cl3—Sn1—C1—C2	86.69 (12)
Cl2—Sn1—N1—C9	43.73 (16)	Cl1—Sn1—C1—C2	177.50 (12)
Cl3—Sn1—N1—C9	−130.99 (16)	Sn1—C1—C2—C3	−78.65 (18)
Cl1—Sn1—N1—C9	138.26 (16)	Sn1—C1—C2—C7	98.78 (16)
N3—Sn1—N1—N2	135.10 (13)	C7—C2—C3—C4	1.1 (2)
Cl2—Sn1—N1—N2	−137.20 (12)	C1—C2—C3—C4	178.60 (16)
Cl3—Sn1—N1—N2	48.08 (12)	C2—C3—C4—C5	−0.1 (3)
Cl1—Sn1—N1—N2	−42.67 (12)	C3—C4—C5—C6	−1.0 (3)
C9—N1—N2—C11	−0.37 (19)	C3—C4—C5—C8	178.73 (17)
Sn1—N1—N2—C11	−179.64 (11)	C4—C5—C6—C7	1.1 (2)
C1—Sn1—N3—C12	−70.61 (15)	C8—C5—C6—C7	−178.67 (17)
N1—Sn1—N3—C12	110.39 (15)	C3—C2—C7—C6	−1.0 (3)
Cl2—Sn1—N3—C12	24.52 (15)	C1—C2—C7—C6	−178.52 (16)
Cl3—Sn1—N3—C12	−165.19 (15)	C5—C6—C7—C2	−0.1 (3)
Cl1—Sn1—N3—C12	122.84 (19)	N2—N1—C9—C10	0.2 (2)
C1—Sn1—N3—N4	99.62 (13)	Sn1—N1—C9—C10	179.44 (13)
N1—Sn1—N3—N4	−79.38 (12)	N1—C9—C10—C11	0.0 (2)
Cl2—Sn1—N3—N4	−165.25 (12)	N1—N2—C11—C10	0.3 (2)
Cl3—Sn1—N3—N4	5.04 (12)	C9—C10—C11—N2	−0.2 (2)
Cl1—Sn1—N3—N4	−66.9 (3)	N4—N3—C12—C13	0.26 (19)
C12—N3—N4—C14	−0.20 (19)	Sn1—N3—C12—C13	171.71 (12)
Sn1—N3—N4—C14	−172.56 (11)	N3—C12—C13—C14	−0.2 (2)
N3—Sn1—C1—C2	−0.09 (13)	N3—N4—C14—C13	0.1 (2)
Cl2—Sn1—C1—C2	−87.78 (12)	C12—C13—C14—N4	0.1 (2)

### Hydrogen-bond geometry (Å, °)

**Table:**

*D*—H···*A*	*D*—H	H···*A*	*D*···*A*	*D*—H···*A*
N2—H2···Cl1^i^	0.87 (1)	2.56 (2)	3.265 (2)	139 (2)
N4—H4···Cl1^ii^	0.88 (1)	2.65 (2)	3.270 (2)	129 (2)

Symmetry codes: (i) −*x*+1, *y*, −*z*+3/2; (ii) *x*, −*y*+1, *z*−1/2.
